# Chemokine Receptor-6 Promotes B-1 Cell Trafficking to Perivascular Adipose Tissue, Local IgM Production and Atheroprotection

**DOI:** 10.3389/fimmu.2021.636013

**Published:** 2021-02-19

**Authors:** Prasad Srikakulapu, Aditi Upadhye, Fabrizio Drago, Heather M. Perry, Sai Vineela Bontha, Chantel McSkimming, Melissa A. Marshall, Angela M. Taylor, Coleen A. McNamara

**Affiliations:** ^1^Carter Immunology Center, University of Virginia, Charlottesville, VA, United States; ^2^Cardiovascular Research Center, University of Virginia, Charlottesville, VA, United States; ^3^Division of Cardiovascular Medicine, Department of Medicine, University of Virginia, Charlottesville, VA, United States

**Keywords:** B-1 cells, IgM, CCR6, atherosclerosis, inflammation, perivascular adipose tissue

## Abstract

Chemokine receptor-6 (CCR6) mediates immune cell recruitment to inflammatory sites and has cell type-specific effects on diet-induced atherosclerosis in mice. Previously we showed that loss of CCR6 in B cells resulted in loss of B cell-mediated atheroprotection, although the B cell subtype mediating this effect was unknown. Perivascular adipose tissue (PVAT) harbors high numbers of B cells including atheroprotective IgM secreting B-1 cells. Production of IgM antibodies is a major mechanism whereby B-1 cells limit atherosclerosis development. Yet whether CCR6 regulates B-1 cell number and production of IgM in the PVAT is unknown. In this present study, flow cytometry experiments demonstrated that both B-1 and B-2 cells express CCR6, albeit at a higher frequency in B-2 cells in both humans and mice. Nevertheless, B-2 cell numbers in peritoneal cavity (PerC), spleen, bone marrow and PVAT were no different in *ApoE*^−/−^*CCR6*^−/−^ compared to *ApoE*^−/−^*CCR6*^+/+^ mice. In contrast, the numbers of atheroprotective IgM secreting B-1 cells were significantly lower in the PVAT of *ApoE*^−/−^*CCR6*^−/−^ compared to *ApoE*^−/−^*CCR6*^+/+^ mice. Surprisingly, adoptive transfer (AT) of CD43^−^ splenic B cells into B cell-deficient μ*MT*^−/−^*ApoE*^−/−^ mice repopulated the PerC with B-1 and B-2 cells and reduced atherosclerosis when transferred into *ApoE*^−/−^*CCR6*^+/+^*sIgM*^−/−^ mice only when those cells expressed both CCR6 and sIgM. CCR6 expression on circulating human B cells in subjects with a high level of atherosclerosis in their coronary arteries was lower only in the putative human B-1 cells. These results provide evidence that B-1 cell CCR6 expression enhances B-1 cell number and IgM secretion in PVAT to provide atheroprotection in mice and suggest potential human relevance to our murine findings.

## Introduction

Atherosclerosis is well-recognized as a chronic inflammatory disease of arteries and plaque rupture is the primary underlying cause of cardiovascular events. Atherosclerosis develops when low density lipoproteins (LDL) enter the subendothelial layer of the artery wall and become oxidized. Products of oxidized lipids are highly reactive and modify self-molecules, thereby generating oxidation-specific epitopes (OSE) that are recognized by receptors of the immune system, including scavenger receptors on macrophages leading to foam cell formation. Oxidized LDL and foam cells promote inflammatory cytokine production and induce the expression of cell adhesion molecules on endothelial cells. Surface adhesion molecule expression recruits inflammatory cells such as monocytes, T cells, natural killer cells, natural killer T cells, and dendritic cells into the subendothelial layer, developing lesion formation (1–3).

B cells play a major role in the regulation of atherosclerosis, and their effects are subset and context-dependent. B-1 B cells attenuate and B-2 B cells aggravate atherosclerosis (4–9). Treatments that deplete predominantly B-2 cells such as anti-CD20 monoclonal antibody and B-cell activating factor receptor (BAFFR) deficiency attenuated atherosclerosis development in apolipoprotein-E deficient (*ApoE*^−/−^) and low-density lipoprotein receptor deficient (*LdlR*^−/−^) mice maintained on Western diet (WD) (5, 6, 8, 9). In contrast, IgM secreting B-1 cells have been shown to be atheroprotective (4, 7). B-1 cells, which are the major source for circulatory IgM (10, 11) and attenuate atherosclerosis (4, 7, 8), can be further divided into two sister populations: B-1a and B-1b. IgM can block the uptake of oxidized LDL by macrophages (12) and, immunization to boost IgM to OSE resulted in atheroprotection (13).

Perivascular adipose tissue (PVAT) directly contacts the artery adventitia and has a role in the regulation of atherosclerosis. Data demonstrates that PVAT adjacent to atherosclerotic human blood vessels is more inflamed than PVAT adjacent to non-diseased vessel segments (14). Adipocytes in PVAT secrete both pro inflammatory and anti-inflammatory cytokines (15). We have recently shown that the PVAT harbors both B-1 and B-2 B cell subtypes in young *ApoE*^−/−^ mice and these local B-1 cells can be induced to proliferate by cytokines (16) and produce atheroprotective IgM antibodies locally (17). However, the mechanism involved in B-1 cell trafficking to PVAT is not known.

Chemokines and chemokine receptors regulate immune cell trafficking and may be key targets or molecules to control homing of immune cells to sites of human disease (18). C-C chemokine receptor 6 (CCR6) is a G protein-coupled receptor expressed on different immune cell types such as macrophages, immature dendritic cells, T and B-lymphocytes (19). CCR6 and its ligand CCL20 have emerged as important regulators of atherosclerosis (18, 20–22). Global deletion of *Ccr6* in *ApoE*^−/−^ and *LdlR*^−/−^ mice resulted in decreased Ly6C^hi^ monocyte exit from the bone marrow, less recruitment of pro-atherogenic macrophages to lesions and attenuated atherosclerosis (20, 22). In human atherosclerosis, the expression of CCR6 and CCL20 in atherosclerotic lesions in the coronary and carotid arteries has been reported. In addition, CCL20 expression is positively correlated with DC numbers in the shoulder regions of the lesion (23), and this DC derived CCL20 may attract CCR6 expressing immune cells into the lesion. Also, circulating levels of CCL20 is significantly increased in hypercholesterolemic patients and LDL stimulated vascular smooth muscle cells express CCL20 and promote human lymphocyte migration (18), implicating a role for the CCR6-CCL20 axis in atherosclerosis development. Previous data from our lab demonstrated that CCR6 regulated aortic homing of CD43^−^ splenocytes (B cells) and diet induced atheroprotection in B cell deficient (μ*MT*^−/−^) mice (21). In general, CD43^−^ splenocytes are thought to be B-2 cells which are considered atherogenic. Thus, it is not clear how the aortic homing of these CD43^−^ splenocytes provides atheroprotection. B cell subsets may have distinct functions in the local PVAT compared to peripheral sites, and atheroprotection may depend on the ability of B cells to home to athero-prone aortic sites (21). Taken together, the results suggest that CCR6 may be a pro- or anti-atherogenic chemokine receptor depending on the cell type in which it is expressed and underscore a need to better understand the role of CCR6 in B cells.

In this current study, we provide the first evidence of a B-1 cell specific function for CCR6 in regulating B-1 numbers and IgM production in PVAT. We demonstrate that atheroprotective effects of CD43^−^ splenocytes in *ApoE*^−/−^*sIgM*^−/−^ mice are CCR6-dependent and require the cells to be capable of secreting IgM implicating PVAT trafficking of B-1 cells as a potentially important atheroprotective mechanism. Further, we demonstrate that in humans, expression of CCR6 on a putative B-1 cell population (24) was significantly reduced in patients with a high degree of coronary artery disease (CAD) underscoring the potential clinical relevance of our findings and suggesting that B-1 cell-specific augmentation of CCR6 expression may be a potential therapeutic approach.

## Materials and Methods

### Animals

All animal protocols were approved by the Animal Care and Use Committee at the University of Virginia. Apolipoprotein E deficient (*ApoE*^−/−^) mice, B cell deficient (μ*MT*^−/−^) mice, and chemokine receptor six deficient (*CCR6*^−/−^) were purchased from Jackson Laboratory and maintained in our animal facility (University of Virginia). μ*MT*^−/−^ and *CCR6*^−/−^ mice were bred to the *ApoE*^−/−^ line to develop *ApoE*^−/−^μ*MT*^−/−^ and *ApoE*^−/−^*CCR6*^−/−^ knockouts. Generated *ApoE*^−/−^*CCR6*^+/−^ mice and setup heterozygous breeders (*ApoE*^−/−^*CCR6*^+/−^ *x ApoE*^−/−^*CCR6*^+/−^) to generate *ApoE*^−/−^*CCR6*^−/−^ and their littermate controls *ApoE*^−/−^*CCR6*^+/+^. All purchased mice were on C57BL/6J background and those bred were backcrossed to C57BL/6J mice for 10 generations. s*IgM*^−/−^ mice were kindly provided by Dr. Peter Lobo (University of Virginia) and crossed to *ApoE*^−/−^ mice to generate *ApoE*^−/−^s*IgM*^−/−^ mice. All mice were given water *ad libitum* and standard chow diet (Tekland, 7012). Mice were euthanized with CO_2_ inhalation. Young (8–10 weeks) male mice were used for all experiments except for atherosclerosis studies. For atherosclerosis studies, *ApoE*^−/−^ mice were maintained on WD (42% fat, Tekland, 88137) for 12 weeks.

### Human Samples

Patients (*n* = 118) were recruited for study through the Cardiac Catheterization laboratory at the University of Virginia as previously described (25). All participants provided written informed consent prior to enrollment, and the study was approved by the Human IRB Committee at UVA. A total of 118 patients presenting for a medically indicated diagnostic cardiac catheterization were enrolled if they met inclusion criteria. Patients were excluded if they had: any acute illness, type 1 diabetes, current acute coronary syndrome, autoimmune disease or on immunosuppressive therapy, prior organ transplantation, anemia, pregnancy, or HIV infection. No patient was on anticoagulation or had deep vein thrombosis or pulmonary embolism. Peripheral blood mononuclear cells (PBMC) were isolated from whole blood for flow cytometry experiments. Isolation of PBMC's from human peripheral blood was performed by RBC lysis in whole blood, and the purified PBMCs were used for flow staining. List of antibodies for cell surface markers (name of the clone) for human flow cytometry: CD3 (SK7 or UCHT1), CD20 (2H7), CD4 (RPA-T4), CD8 (RPA-T8), CD14 (61D3), CCR6 (11A9), CD27 (M-T271) and CD43 (1G10) were purchased from eBioscience and BD Bioscience. Live/Dead discrimination was determined by LIVE/DEAD fixable yellow staining (Invitrogen). Cells were run on a CyAN ADP (Beckman Coulter). Data were analyzed with FlowJo 9 software.

### Coronary Artery Disease Severity Measured by Gensini Score

Gensini Score (GS) is a widely used angiographic scoring system to measure the severity of coronary artery disease (CAD) (26). We used quartiles to categorize patients with CAD based on GS. Participants with scores in the first three quartiles (GS: 0–33.25) were categorized in the low GS group (*n* = 80) and participants with scores in fourth quartiles (GS: 33.25–128) were categorized as in the high GS group (*n* = 38). Quartile values were calculated using a larger cohort in which the current cohort is nested.

### Flow Cytometry

Peritoneal cavity lavage (PerC), spleen and bone marrow (BM) cells were harvested and single cell suspensions were prepared as previously described (27). In brief, cell suspension from spleen was prepared using a 70 μm cell strainer and mashing spleen with a syringe plunger, and dissolved in FACS buffer. To isolate BM cells, femur and tibia were collected and flushed with FACS buffer. Spleen and BM samples were re-suspended in erythrocyte lysis buffer and washed. Stromal vascular fraction was prepared from PVAT as previously described (27). In brief, to harvest PVAT, first, para aortic lymph nodes were carefully removed and then PVAT was carefully harvested and weighed. PVAT was collected into 5 mL FACS tubes separately, 2 mL of freshly prepared enzyme cocktail mixture [Collagenase I (450 U/ml) (Sigma), Collagenase XI (125 U/ml) (Sigma), Hyaluronidase I (60 U/ml) (Sigma), DNase (60 U/ml) (Sigma) in PBS with 20 mM HEPES] was added per sample. PVAT was chopped into small pieces and then incubated in a shaking incubator at 37°C for 45 min to obtain single cell suspensions.

For flow staining, cells were blocked for Fc receptors by Fc block (CD16/32) for 10 min on ice, and were stained for cell surface markers using fluorescently conjugated antibodies for 30 min on ice. After washing and centrifugation, cells were washed and stained with a fixable live/dead stain diluted in PBS for 15 min on ice and then fixed in 2% PFA in PBS for 10 min at room temperature prior to re-suspending in FACS buffer. Flow cytometry antibodies: CD19 (1D3), B220/CD45R (RA3-6B2), CD5 (53-7.3), CD43 (S7), CCR6 (29-2L17) and IgM (II/41, R6-60.2) were purchased from eBioscience, BD Bioscience, and Biolegend. Live/Dead discrimination was determined by LIVE/DEAD fixable yellow staining (Invitrogen). Cells were run on CyAN ADP (Beckman Coulter) and Attune NxT flow cytometer (Invitrogen). Data were analyzed with FlowJo 10 software.

### Adoptive Transfer Experiments

PerC cells were isolated from *ApoE*^−/−^ mice and stained for FACSorting (InFlux sorter). Cells were stained with fluorescence labeled antibodies against CD19 (1D3), B220 (RA3-6B2), CD23 (B3B4), IgD (11-26c) and CD5 (53-7.3), and live cells were gated for DAPI^−^ population. Two hundred thousand of FACSorted B-1 cells were adoptively transferred into 8–10 week old *ApoE*^−/−^s*IgM*^−/−^ mice via intraperitoneal injection. For CD43^−^ splenocyte isolation, spleens were harvested from 10–12 week old donor mice and B cells were isolated using MACS anti-CD43 microbeads (Miltenyi Biotec) as per the manufacturer's protocol. The purity of representative samples was analyzed by flow cytometry and found to be >97%. Either 30 × 10^6^ or 60 × 10^6^ B cells were resuspended in 200 μl of PBS and adoptively transferred *via* tail vein injection to 8–10 week old recipients.

### Enzyme-Linked ImmunoSpot Assay

Single cell suspensions of PVAT, spleen and BM were prepared as described above in the flow cytometry section. ELISPOT was performed as previously described (4, 17, 27). Sterile MultiScreen IP-Plates (Millipore, MSIPS4510) were used for the assay according to manufacturer's protocol. Wells were coated with unlabeled goat anti-mouse IgM antibody (10 μg/ml; Southern Biotech) and incubated overnight at 4°C. The next day, antibody solution was decanted, membrane was washed with PBS and then blocked with RPMI 1640+10% FCS for 2 h at 37°C. A suspension of 1 × 10^6^ cells/ml was prepared in ice cold culture media for spleen and BM from which 100,000 cells were plated for each of the sample as starting concentration and then were serially diluted in subsequent wells. For PVAT samples, resuspended in 250 μl culture media and were used as starting concentration from which serial dilutions in subsequent wells were prepared. The plate was incubated overnight at 37 °C in a cell culture incubator (5% CO_2_). Cells were decanted, washed (PBS+0.01% tween-20) and incubated with biotin-labeled goat anti-mouse IgM antibody (1:500 dilution) (Southern Biotech) for 2 h in a cell culture incubator. After washing, cells were incubated for 30 min at room temperature in streptavidin alkaline phosphatase (Abcam). Again, following washing BCIP/NBT (Gene Tex Inc.) was added and incubated until spots became visible. Each spot on the membrane indicated an antibody secreting cell. Spots were counted manually.

### Enzyme-Linked Immunosorbent Assay

Plasma or serum samples were collected from mice and circulatory total IgM and IgG levels were quantified by ELISA as published before (28). Total IgE ELISA was performed according to manufacturer's guidelines (BioLegend). For MDA epitope specific IgM and IgG ELISA, we used Peptide mimotope for malondialdehyde (MDA) epitope (P2 peptide-biotin) (Peptide 2.0) as a capture antigen (29). Briefly, 96 well microtiter plates (Corning) were incubated at 4°C overnight with capture un labeled IgM or IgG diluted in coating buffer (0.1 M disodium phosphate pH 9.0). Plates were blocked (PBS containing 0.5% BSA, 0.1% TWEEN-20, and 0.01% NaN_3_), incubated with samples, and then treated with IgM detection antibody conjugated to alkaline phosphatase for 2 h at room temperature. Detection antibodies and dilutions used: murine IgM-AP and murine IgG-AP (Southern Biotech). Plates were then developed with pNPP solution (Southern Biotech) for 30–60 min and read at 405 nm using a SpectraMax 190 (Molecular Devices). IgM concentration was determined through a standard curve of purified immunoglobulin (Southern Biotech) using a range of 0.098–200 ng/ml. All dilutions were determined through careful titration, and only values within the range of standard curves with readings at least 3-fold higher than negative controls were used.

### Enface Staining

Aortas were harvested carefully as previously described (17). Aortas were opened longitudinally, fixed in 4% formaldehyde, pinned, and stained with Sudan IV (Sigma). Aortas were imaged with a Nikon D70 DSLR camera and enface lesion area was quantified using ImagePro Plus 7.0 software.

### Statistics

Student's *t*-test was used for analyzing data with normal distribution and equal variance. For data sets with non-normal distribution, Wilcoxon rank-sum test was used. Similarly, a non-parametric test (Spearman's correlation) was used to correlate data that was not normally distributed. One-way Anova with multiple comparison was used when compared multiple groups. Results are displayed containing all replicated experiments, and values shown are mean ± SEM. Data were analyzed using Prism 8 (GraphPad Software, Inc) and SAS (SAS version 9.4).

## Results

### A Higher Frequency of CCR6^+^ B-1 Cells in Tissue Compartments That Support Antibody Production Compared to Their Homeostatic Niche

**Figure 1:**
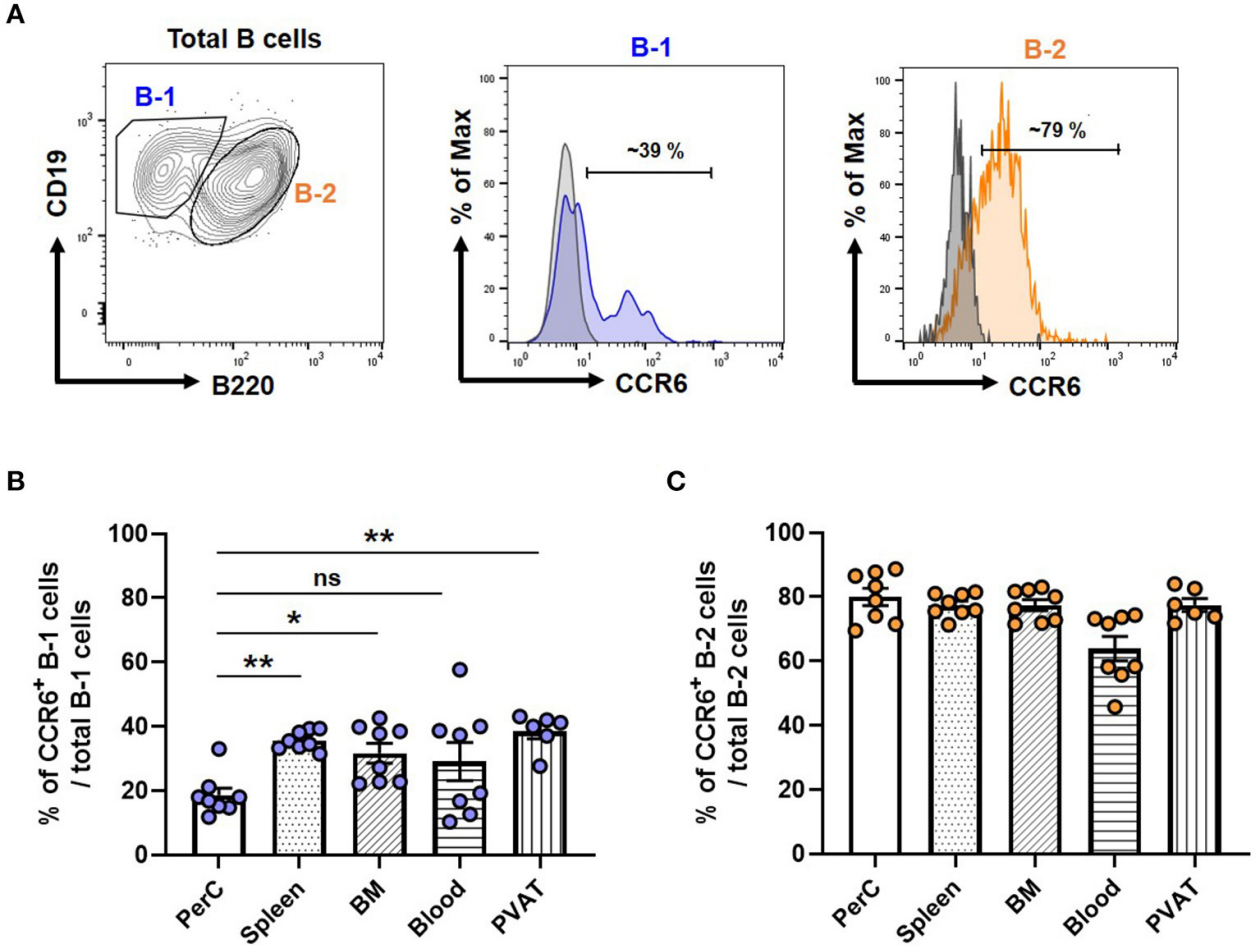
Frequency of CCR6^+^ B-1 cells was higher in antibody secreting tissue compartments in mice. **(A)** Gating strategy for B-1 and B-2 cells in PVAT and representative histogram of CCR6 expression. Gray line represents FMO for CCR6 and filled blue line represents B-1 or filled orange line represents B-2 cells in flow cytometry histogram plots **(B)** percentage of CCR6^+^ B-1 cells from total B-1 cells and **(C)** percentage of CCR6^+^ B-2 cells from total B-2 cells in different tissue compartments. Results are represented in mean ± SEM, Performed One-way ANOVA with multiple comparisons. *p < 0.05 and **p < 0.01. *n* = 6–8 mice/group and each dot represents individual mouse.

To compare the expression levels of CCR6 in B-1 and B-2 cells, we performed flow cytometry on isolated cells from various compartments and measured the frequency of CCR6^+^ B-1 and B-2 cells from atherosclerosis prone chow fed *ApoE*^−/−^ mice. Figure 1A depicts the gating strategy for identifying B-1 and B-2 cells and a representative histogram showing CCR6 expression relative to an FMO control. Quantification of the frequency of CCR6^+^ B-1 and B-2 cells in different tissue compartments revealed that both B-1 and B-2 cells express CCR6. Interestingly, the frequency of CCR6^+^ B-1 cells was significantly higher in antibody secreting tissue compartments such as spleen, BM and PVAT compared to the primary B-1 cell niche, PerC (Figure 1B). However, despite a higher frequency of CCR6^+^ cells, there was no such difference observed in B-2 cells (Figure 1C). This data suggests that CCR6 may be important for B-1 cell recruitment to antibody secreting regions.

### CCR6 Deficiency Reduces B-1 Cell Numbers in PVAT

**Figure 2:**
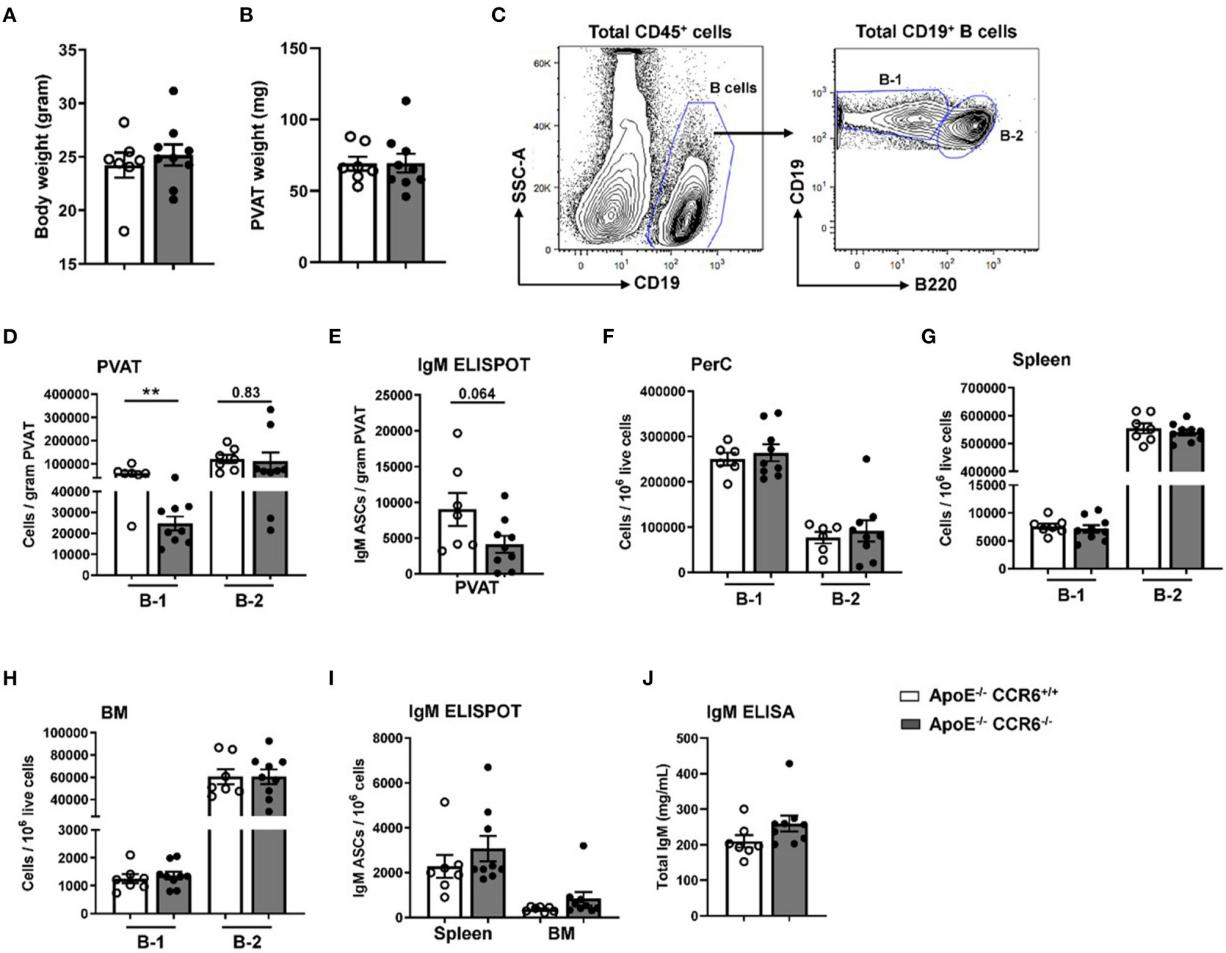
IgM secreting B-1 cells were reduced in PVAT of *ApoE*^−/−^ *CCR6*^−/−^ mice. Comparison of *ApoE*^−/−^*CCR6*^+/+^ and *ApoE*^−/−^*CCR6*^−/−^ littermate control mice showed no difference in **(A)** body weight or **(B)** PVAT weights between groups. **(C)** Gating strategy for total B cells, B-1 and B-2 cells in PVAT. Quantification of **(D)** total B-1 and B-2 cells and **(E)** IgM secreting cells in PVAT. **(F–H)** Quantification of total B-1 and B-2 cells in PerC **(F)**, spleen **(G)** and BM **(H)** and IgM secreting cells in **(I)** spleen and BM. **(J)** quantification of total IgM levels in plasma. Results are represented in mean ± SEM, unpaired student *t*-test was performed. **p < 0.01. *n* = 7–9 mice/group and each dot represents individual mouse.

Previous data from our lab clearly showed that CCR6 regulates B cell migration to aorta and provides atheroprotection (21) and that PVAT harbors higher numbers of atheroprotective IgM-producing B-1 cells than the aorta itself (17). To determine the effect of CCR6 deficiency on B cell subset distribution in PVAT and other tissue compartments in atherosclerosis-prone mice at homeostasis, flow cytometry and ELISPOT experiments were performed in young *ApoE*^−/−^*CCR6*^+/+^ and *ApoE*^−/−^*CCR6*^−/−^ littermate control mice (10 weeks old) fed normal Chow diet. There was no difference in total body weight (Figure 2A) and PVAT weight (Figure 2B). PVAT collected from the aortic arch to the iliac bifurcation was carefully dissected and flow cytometry was performed to analyze B-1 and B-2 cells (Figure 2C). Flow cytometry data revealed B-1 cell numbers but not B-2 cell numbers were significantly reduced in PVAT of *ApoE*^−/−^*CCR6*^−/−^ mice compared to *ApoE*^−/−^*CCR6*^+/+^ mice (Figure 2D). ELISPOT data demonstrate that total IgM secreting B cells were reduced in PVAT in *ApoE*^−/−^*CCR6*^−/−^ mice compared to *ApoE*^−/−^*CCR6*^+/+^ mice (Figure 2E). Intriguingly, there was no difference of B-1 and B-2 cell numbers in PerC, spleen and BM compartments between *ApoE*^−/−^*CCR6*^+/+^ and *ApoE*^−/−^*CCR6*^−/−^ mice (Figures 2F–H). Correspondingly, there was no difference in total IgM secreting B cells in spleen and BM *ApoE*^−/−^*CCR6*^+/+^ and *ApoE*^−/−^*CCR6*^−/−^ mice (Figure 2I), and there was no difference in plasma total IgM levels between *ApoE*^−/−^*CCR6*^+/+^ and *ApoE*^−/−^*CCR6*^−/−^ mice as well (Figure 2J).

### CD43^–^ Splenocytes (B cells) Can Repopulate/Establish B-1 Cell Compartment

To understand whether B-1 cells can migrate, survive and produce IgM in PVAT, FACS-sorted B-1 cells (2 × 10^5^) were adoptively transferred into secretory IgM (*sIgM*^−/−^) deficient *ApoE*^−/−^ mice via i.p injection. After 7 weeks of the adoptive transfer, plasma IgM levels were measured by ELISA at different time points. The plasma IgM levels were boosted after 1st week of the B-1 cell transfer compared to PBS injected *ApoE*^−/−^*sIgM*^−/−^ mice. However, secreted IgM levels in the plasma eventually dropped down to baseline within 4 weeks of transfer (Supplementary Figure 1). Flowcytometry analysis confirmed non-survival of adoptively transferred B-1 cells in *ApoE*^−/−^*sIgM*^−/−^ mice after 2 weeks (data not shown). Since survival of B-1 cells alone is not possible for long-term experiment we sought out an alternative approach.

**Figure 3:**
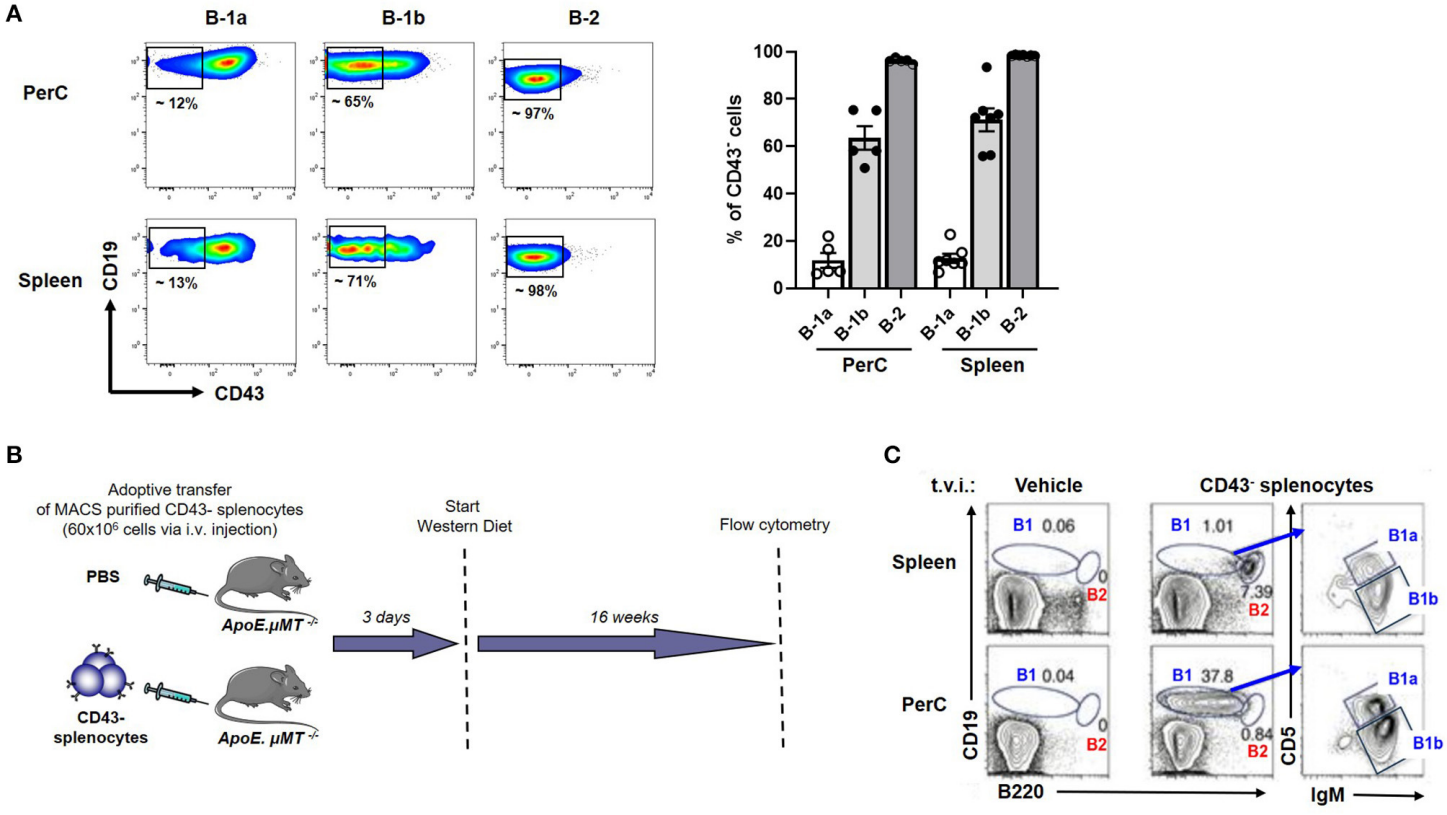
CD43^−^ splenocytes (B cells) contains a CD43^−^ B-1 compartment, predominantly B-1b. **(A)** flow cytometric analysis for CD43^+^ B-1a, B-1b and B-2 cells in PerC and spleen of young *ApoE*^−/−^ mice (*n* = 5–7). Each dot represents individual mouse. **(B)** Schematic representation of experimental design. CD43^−^ splenocytes from *ApoE*^−/−^ mice or vehicle control were tail vein injected (t.v.i.) into μ*MT*^−/−^*ApoE*^−/−^ mice. **(C)** CD43^−^ splenocytes reconstituted PerC B-1 cells and splenic B-2 cells in μ*MT*^−/−^*ApoE*^−/−^ mice.

We previously showed that adoptive transfer of CD43^−^ splenocytes (B cells) into B cell deficient mice provided atheroprotection compared to vehicle (PBS) injected mice (21). Yet, CD43^−^ splenocytes have been thought to represent B-2 cells which are considered atherogenic (5, 6, 8, 9) and B-1 cells are CD43^+^ (10, 17, 30). However, this paradigm did not explain how CD43^−^ B cell transfer provided atheroprotection in B cell deficient *ApoE*^−/−^ mice (*ApoE*^−/−^μ*MT*^−/−^) after 16 weeks of WD (21), raising an important question whether there could be B-1 cells in the CD43^−^ compartment. We analyzed CD43 expression in B cell compartments by doing flow cytometry in *ApoE*^−/−^ mice. Flow data clearly demonstrate that the majority (>95%) of B-2 cells and ~10-15 % of B-1a were CD43^−^ in both PerC and spleen. Interestingly, ~60% of B-1b cells were CD43^−^ in both PerC and spleen (Figure 3A). The data suggested that the CD43^−^ splenocyte population contains remnant B-1 cells, in particular B-1b cells. Next, to address whether CD43^−^ splenocytes can repopulate B-1 cells in recipient mice, MACS purified CD43^−^ splenocytes (B cells; 60 × 10^6^) were adoptively transferred via tail vein injection into *ApoE*^−/−^μ*MT*^−/−^ mice and maintained on WD for 16 weeks (Figure 3B). Interestingly, flow cytometry data clearly demonstrated that adoptively transferred CD43^−^ splenocytes could repopulate both B-1 and B-2 cells in PerC and spleen respectively in *ApoE*^−/−^μ*MT*^−/−^ mice. In addition, these B-1 cells contain both B-1a and B-1b subsets (Figure 3C). Also, flow cytometry data confirmed that the purity of CD43^−^ splenocytes after MACS separation was >97%. MACS separation significantly reduced CD43^+^ B-1 compartment (~ 90 %) (Supplementary Figure 2). It is also possible that the remnant CD43^+^ cells could also repopulate. We next wanted to determine if these CD43^−^ splenocytes in the absence of CCR6 can provide IgM mediated atheroprotection.

### Atheroprotection by IgM Secreting B Cells Is CCR6 Dependent

**Figure 4:**
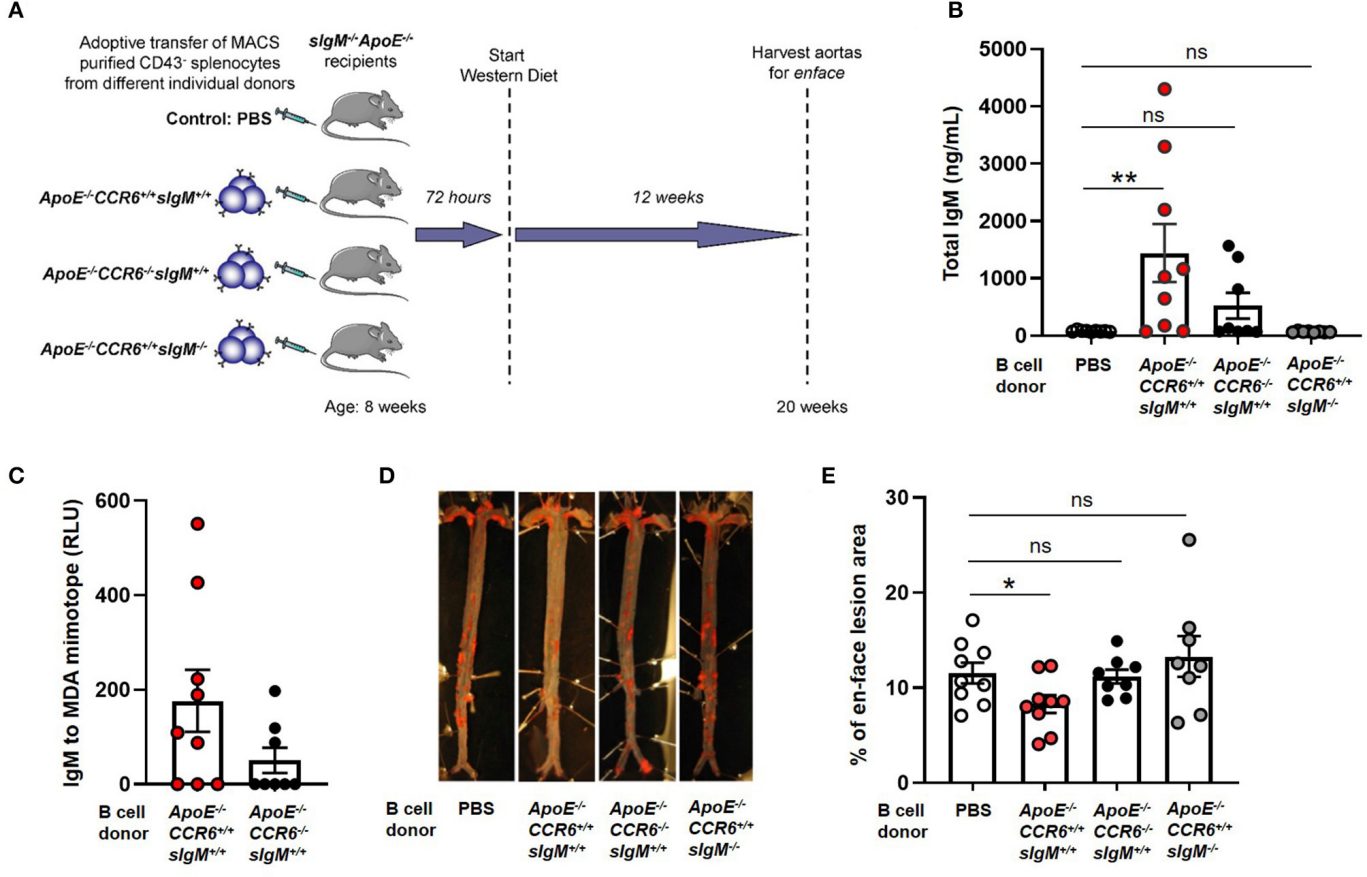
CCR6 expression on IgM secreting B cells is important to provide atheroprotection. **(A)** Schematic representation of experimental design. **(B)** quantification of plasma IgM levels, **(C)** MDA mimotope specific IgM, **(D)** *en-face* staining of aortas and **(E)** quantification of atherosclerosis levels in *sIgM*^−/−^ *ApoE*^−/−^ mice after adoptive transfer of CD43^−^ splenocytes from different genotypic donor mice followed by 12 weeks of WD feeding. Results are represented in mean ± SEM, Mann-Whitney unpaired *t*-test was performed. *p < 0.05, **p < 0.01. *n* = 8–9 mice/group and each dot represents individual mouse.

To determine the effect of CCR6 on IgM secreting B cell mediated atheroprotection, 30 × 10^6^ CD43^−^ splenocytes from *ApoE*^−/−^*CCR6*^+/+^*sIgM*^+/+^ or *ApoE*^−/−^*CCR6*^−/−^*sIgM*^+/+^ or *ApoE*^−/−^*CCR6*^+/+^*sIgM*^−/−^ or PBS (control) mice were adoptively transferred via tail vein injection into *ApoE*^−/−^*CCR6*^+/+^*sIgM*^−/−^ mice following which the recipients were fed WD for 12 weeks (Figure 4A). Cholesterol levels were no different between the groups after 12 weeks of WD (Supplementary Figure 3). We started by determining the effect of CCR6 on IgM production. We measured circulatory IgM levels by ELISA. Interestingly, circulatory IgM levels were significantly higher in mice that received B cells from *ApoE*^−/−^*CCR6*^+/+^*sIgM*^+/+^ mice but not in mice that received CD43^−^ splenocytes from *ApoE*^−/−^*CCR6*^−/−^*sIgM*^+/+^ mice compared to PBS (control) mice. While these differences are detectable in *sIgM*^−/−^ mice, these levels are far lower than in WT mice, likely obscuring this small but significant difference. As expected, there was no difference of circulatory IgM levels in mice that received CD43^−^ splenocytes from *ApoE*^−/−^*CCR6*^+/+^*sIgM*^−/−^ mice compared to PBS (control) mice (Figure 4B). A trend of lower levels of circulatory IgM and MDA (oxidation specific epitope) mimotope specific IgM were observed in recipients given *ApoE*^−/−^*CCR6*^−/−^*sIgM*^+/+^ CD43^−^ splenocytes, compared to those given *ApoE*^−/−^*CCR6*^+/+^*sIgM*^+/+^ CD43^−^ splenocytes (Figures 4B,C). No difference in circulatory total IgG, IgE and MDA mimotope specific IgG were observed between PBS (control) and B cell recipient groups (Supplementary Figure 4). Next, we wanted to measure atherosclerosis levels. The percentage of enface lesion area was quantified in aortas by Sudan IV staining (Figure 4D). Intriguingly, atherosclerosis levels were significantly reduced in mice that received CD43^−^ splenocytes from *ApoE*^−/−^*CCR6*^+/+^*sIgM*^+/+^ mice compared to those that received PBS (control). There was no difference in atherosclerosis levels in mice that received CD43^−^ splenocytes from *ApoE*^−/−^*CCR6*^−/−^*sIgM*^+/+^ or *ApoE*^−/−^*CCR6*^+/+^*sIgM*^−/−^ compared to PBS (control). Moreover, the CD43^−^ splenocytes from *ApoE*^−/−^*CCR6*^+/+^ mice attenuated atherosclerosis only when they were capable of secreting IgM (Figure 4E).

### Expression of CCR6 on Human Putative B-1 Cells (CD20^+^ CD27^+^ CD43^+^) Was Significantly Lower in Patients With High CAD Severity Scores

**Figure 5:**
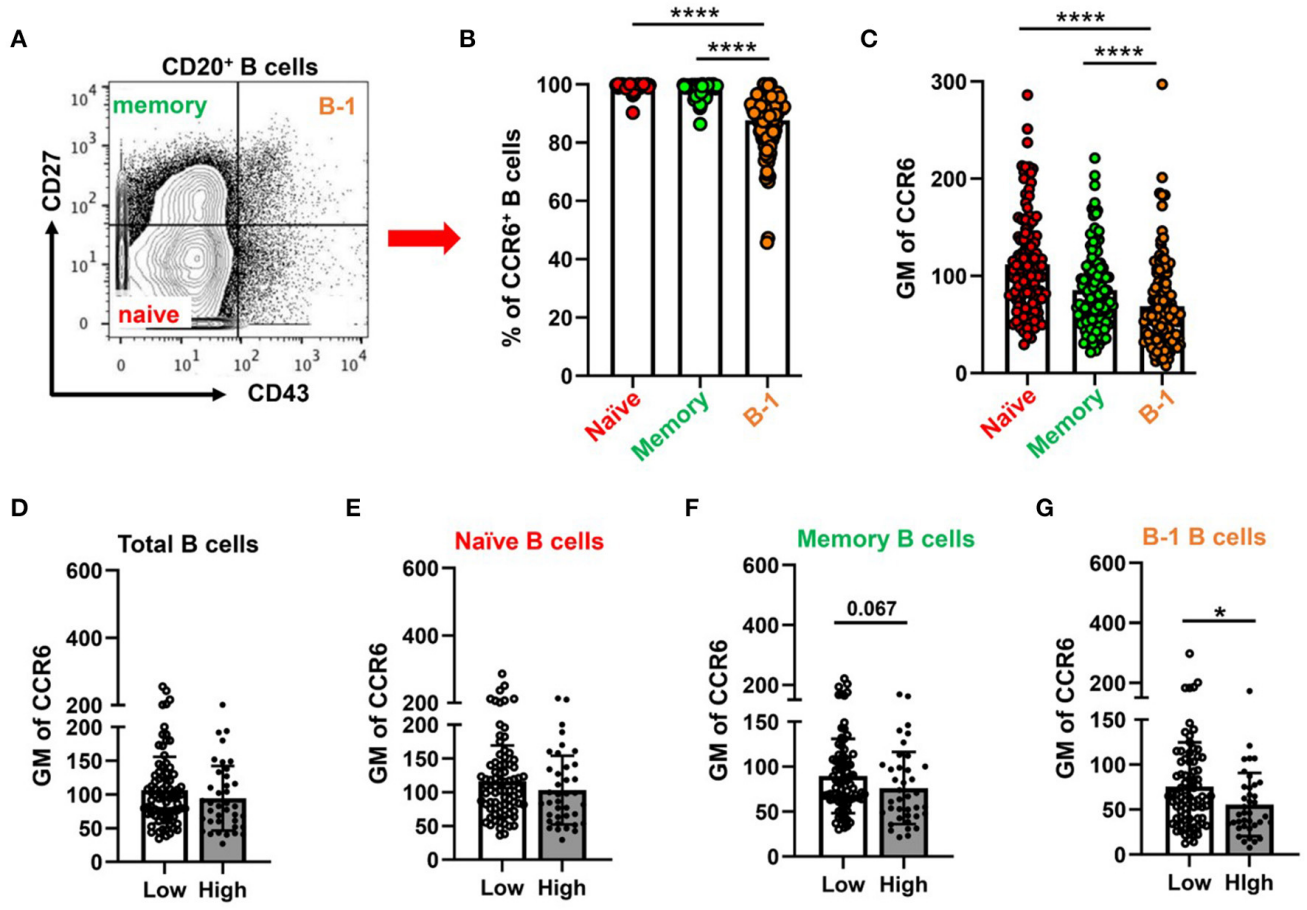
Percentage of CCR6^+^ and GM of CCR6 on human circulating B cell subtype with lower CCR6 GM on putative human B-1 cells from subjects with high CAD severity score. **(A)** gating strategy for circulating B cell subsets (naïve, memory and B-1 B cells) by using CD43 and CD27. **(B)** percentage of CCR6^+^ B cell subsets from total B cells and **(C)** GM of CCR6 expression on B cell subsets. Results are shown in mean ± SEM, paired *t*-test was performed. ****p < 0.0001. **(D–G)** GM of CCR6 on total B cells **(D)**, naïve **(E)**, memory **(F)** and B-1 B cells **(G)** in subjects with low and high Gensini scores. Results are shown in mean ± SEM, Mann-Whitney *t*-test was performed. *p < 0.05.

Next, to examine CCR6 expression on circulatory B cell subsets including putative B-1 cells in humans, PBMCs were collected from patients presenting for a medically indicated coronary angiography CAD assessment. Flow cytometry was performed on PBMCs with antibodies that allowed quantification of CCR6 on B cells, T cells and monocytes. Consistent with previous reports (31–34), all B cells express CCR6 and ~27% of CD4^+^ T cells and ~ 10% of CD8^+^ T cells express CCR6. However, circulating human monocytes do not express CCR6 (Supplementary Figure 5), despite CCR6 on monocytes being implicated in atherosclerosis in murine models (20, 22). Next to understand the CCR6 expression levels on B-1 cells, total CD20^+^ B cells were gated for naïve, memory and B-1 cells by using CD43 and CD27 (Figure 5A) (24, 35). The frequency of CCR6^+^ B-1 cells (Figure 5B) and GM mean of CCR6 expression on CD20^+^CD27^+^CD43^+^ B-1 cells (Figure 5C) was lower compared to naïve and memory B cells in circulation.

To determine whether expression of CCR6 on circulating B cell subtypes was associated with the severity of CAD, we utilized the well-established gensini scoring system (GS) as outlined in the section Methods. GM of CCR6 on total B cells and B cell subsets was quantified in low GS and high GS patients. There was no difference in age, gender, diabetes and hypertension between GS low and high groups (Supplementary Table 1). There was no difference of GM of CCR6 on total B cells, naïve and memory B cells (Figures 5D–F). Interestingly, GM of CCR6 on B-1 cells was significantly lower in patients with high GS (Figure 5G), suggesting that CCR6 expression on human B-1 cells may protect from development of severe coronary artery atherosclerosis.

## Discussion

The abundance of atheroprotective B-1 cells in the PVAT has been previously reported by our group (17). However, the mechanism and the role of chemokine receptors in their homing to the PVAT has not been demonstrated. Chemokine receptors are important for the recruitment or migration of immune cells to lymphoid tissues and inflammatory sites to regulate the immune responses. CCR6 is expressed on different leukocyte populations, such as immature dendritic cells (36), B cells (32, 33), T cells (34), NKT cells (37). Previous studies of CCR6 on B cells mainly focused on mature adaptive B-2 cells. Our study is the first to identify a role for CCR6 in regulating B-1 cell number and IgM production in PVAT and a CCR6-dependent IgM-mediated inhibition of diet-induced atherosclerosis.

Two decades ago, an elegant study was published by Krzysiek et al., where they demonstrated the functional role of CCR6 expression during B cell development and antigen mediated B cell differentiation in humans. CCR6 is not expressed during early B cell development in the BM. However, it is expressed later by all mature B cells in the BM, peripheral blood and umbilical cord blood. Eventually, mature B cells lose their CCR6 expression upon activation via B cell receptor signaling and entering into germinal center reactions and terminally differentiating into antibody secreting plasma cells in secondary lymphoid organs. Interestingly, CCR6 is re-expressed once these germinal center B cells differentiate into memory B cells (33) and also these cells show increased chemotactic response to the CCR6 ligand, CCL20 (38). This data suggests that CCR6 expression is restricted to functionally mature B cells capable of responding to antigen challenge. Another study demonstrated that CCR6 expression was important for the migration of memory B cells to the mucosal tissue to produce IgA against intestinal microbial antigens (39). CCR6 is necessary for positioning of memory B cells in spleen to mount recall responses to the same antigen (40) and is thus involved in the regulation of B cell development and their function, particularly, mature activated B-2 cells.

Consistent with previous reports (31, 33), our flow cytometry data in human PBMCs showed that almost all B cells, a small percentage of T cells, and none of the monocytes in circulation express CCR6. In addition, our human data shows that the frequency of CCR6^+^ B-1 cells was significantly lower compared to naïve, and memory B cells in circulation. Though the frequency of CCR6^+^ B-1 cells was lower than B-2 cells in all tissue compartments in the murine system at homeostasis, the frequency of CCR6^+^ B-1 cells in antibody secreting tissue compartments such as the spleen, BM and PVAT was higher compared to their primary homeostatic niche (PerC). Further, in the absence of CCR6 (*ApoE*^−/−^*CCR6*^−/−^) there were significantly fewer IgM secreting B-1 cells in PVAT but not in PerC, spleen and BM. This data suggests that CCR6 expression on B-1 cells may play an important role in B-1 recruitment to PVAT to attenuate disease progression.

Our murine data showed that the number of atheroprotective IgM secreting B-1 cells were significantly reduced in PVAT of *ApoE*^−/−^*CCR6*^−/−^ compared to control group at homeostasis. However, there is no difference in plasma IgM levels between *ApoE*^−/−^*CCR6*^+/+^ and *ApoE*^−/−^*CCR6*^−/−^. This may be due to lack of differences in B-1 cell numbers in the major IgM producing sites such as spleen and BM. Similar to the role of chemokine receptors such as CXCR5, which is important for recruitment/migration of B-1 cells to their primary niches such as PerC and omental fat (41) and CXCR4 in regulation of atheroprotective IgM secreting B-1a cell recruitment to bone marrow (35), our findings provide evidence that CCR6 is important for B-1 cell recruitment to PVAT. The atheroprotective effect is likely due to local (PVAT) IgM secretion, not detected by changes in plasma levels.

Murine studies demonstrate that CCR6 on monocytes is important for their recruitment to aorta to aggravate atherosclerosis. In CCR6 deficient *ApoE*^−/−^ mice atherosclerosis levels were significantly reduced compared to littermate controls (*ApoE*^−/−^*CCR6*^+/+^) after WD feeding which can be attributed to reduction in BM derived Ly6C^hi^ inflammatory monocyte subtype in circulation and thereby reduction in macrophage numbers in atherosclerotic lesions (20). A similar finding was reported in CCR6 deficient *LdlR*^−/−^ mice where atherosclerosis levels were significantly reduced in aorta and aortic sinus *via* reduction in the numbers of Gr-1^hi^ and Gr-1^low^ monocytes in circulation followed by reduction in accumulation of macrophage numbers in the lesions when compared to *LdlR*^−/−^*CCR6*^+/+^ control mice. Contrary to the changes observed in monocyte subset numbers, there was no difference in frequencies of Th1, Th17 and regulatory T cells locally and systemically between *LdlR*^−/−^*CCR6*^−/−^ and *LdlR*^−/−^*CCR6*^+/+^ mice (22). Consistent with our human data on CCR6 expression on human leukocytes, initially, CCR6 was not thought to be expressed on human monocytes (42). However, studies have shown a substantial increase in CCR6 expression on monocytes during inflammatory conditions. Around 3% of circulatory monocytes expressed CCR6 in blood and synovial fluid from rheumatoid arthritis patients and these monocytes responded to CCL20 chemotaxis *in vitro* (31). This suggests that chemokine receptor expression may increase on leukocytes during inflammatory conditions and promote recruitment to sites of inflammation in humans.

While global deletion of CCR6 conferred atheroprotection, previous studies of the role of CCR6 on B cells demonstrate an atheroprotective effect. Adoptive transfer of CD43^−^ splenocytes from CCR6^+/+^ but not CCR6^−/−^ donors showed increased recruitment of B cells to the aorta (taken together with PVAT) and significantly reduced diet induced atherogenesis in μ*MT*^−/−^ recipients (21). Also, upon comparing aorta and PVAT separately, we have shown that PVAT and not aorta has more B cells (17). This suggests that CCR6 is involved in B cell homing to PVAT and in conferring B cell-mediated atheroprotection. Adoptive transfer of FACS sorted B-1 cells alone do not survive long-term in *ApoE*^−/−^*sIgM*^−/−^ recipients. We therefore took the approach of transferring CD43^−^ splenocytes as per our previous publication (21). Notably, although the percentage of splenic B-1 cells that are CD43^−^ is lower than the percentage of CD43^−^ B-2 cells, 10-15% of B-1a and 60-70% of B-1b cells in both PerC and spleen are CD43^−^ (Figure 3A). These B-1 cells together with few remnant CD43^+^ B-1 cells could re-populate during the 16 weeks of time prior to the end of our study due to their self-renewing capacity. Our current study suggests that this re-population of atheroprotective IgM secreting B-1 cells led to atheroprotection.

In this current study we performed the adoptive transfer of CD43^−^ splenocytes into sIgM deficient mice that have endogenous B cells unlike the *uMT*^−/−^ mice in our previous study (21). sIgM deficiency increases marginal zone B cells and reduces follicular B cells (43, 44). Though marginal zone B cells are primarily involved in providing protection from blood borne pathogens (45). However, these B cells are also involved in atheroprotection by reducing follicular T helper cell development via programmed death ligand-1 signaling (46). These follicular T helper cells are important for differentiation of follicular B cells into germinal center B cells followed by antigen specific antibody producing B cells. IgG is a major antibody type secreted by B cells that undergo germinal center reactions. With regards to follicular B cells, it has been reported that *sIgM*^−/−^ mice display increased B cell receptor signaling that results in abnormal B cell development followed by reduced follicular B cell numbers (44). This condition leads to increased circulatory pathogenic IgE levels in circulation and aggravated atherosclerosis in atherogenic diet fed *Ldlr*^−/−^*sIgM*^−/−^ mice compared to control group (47). None of the *ApoE*^−/−^*sIgM*^−/−^ recipient mice in our study regardless of genotype of the donor cells had differences in circulatory total IgG and IgE antibody levels arguing against this mechanism for the atheroprotection seen in our study.

**Figure 6:**
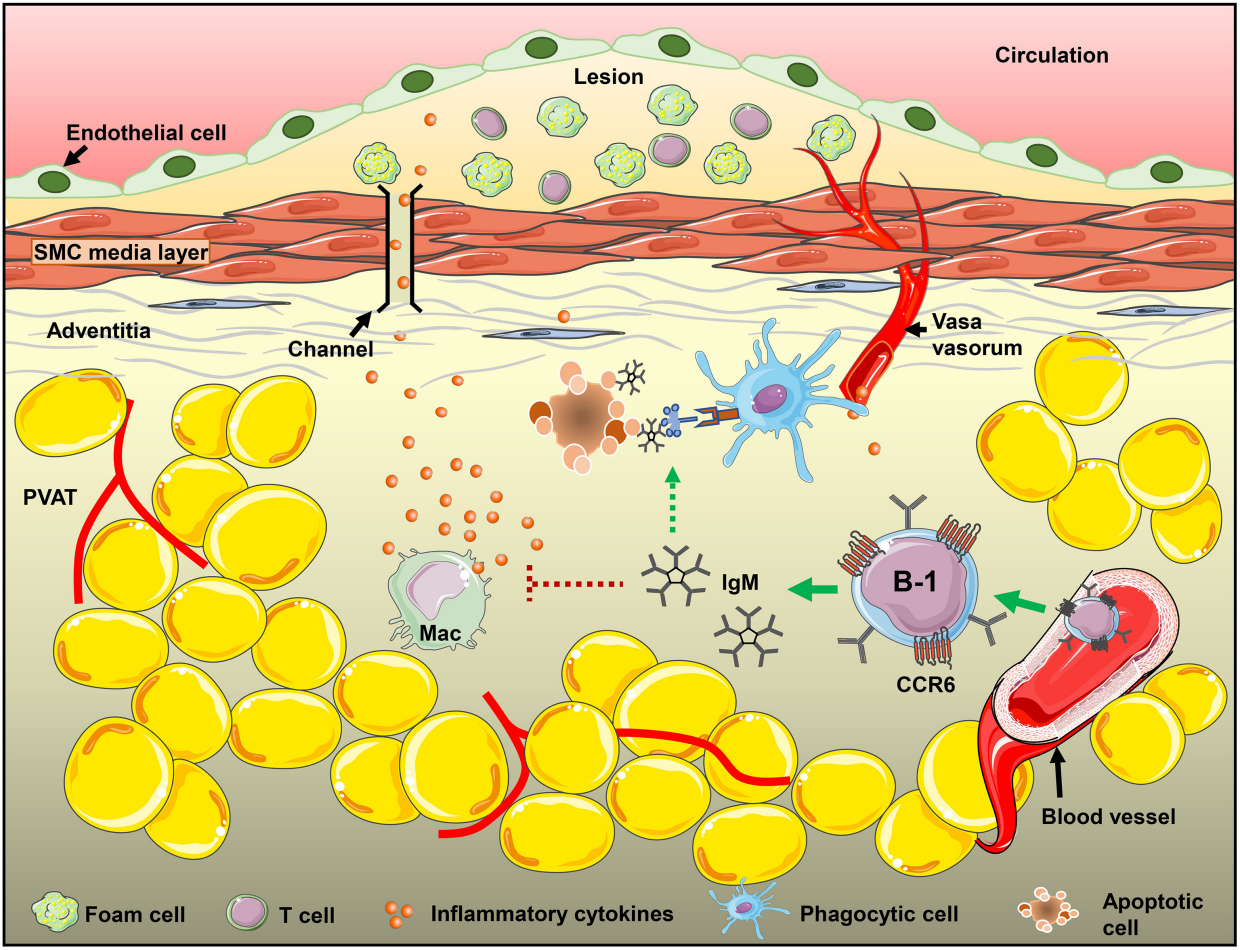
CCR6 expression on B-1 cells regulate B-1 numbers in PVAT and these B-1 cells attenuate diet-induced atherosclerosis in an IgM-dependent manner via inhibiting inflammatory cytokine production from inflammatory macrophages and/or increasing apoptotic cell clearance in the PVAT.

We have previously shown that sIgM deficiency aggravates atherosclerosis in mice (48) and local PVAT mRNA levels of proinflammatory cytokines like INFγ and TNFα in *sIgM*^−/−^ mice are significantly higher compared to littermate controls (unpublished data). This supports the role of locally produced IgM in regulation of PVAT inflammation. This data is consistent with our previous report that show that CCR6 deficiency significantly reduced B cell recruitment to aorta and adoptive transfer of B cells from *CCR6*^−/−^ mice no longer provided atheroprotection (21). CCR6 is thus important for B-1 cell recruitment and local IgM production in the PVAT. B-1 cell derived IgM in fact could influence the disease levels by altering the local environment in the PVAT of mice either by reducing inflammatory cytokine production (TNFα and MCP-1) from M1 type macrophages in adipose tissue (28), or by mediating opsonization of apoptotic cells and accelerating their clearance by phagocytic cells (49, 50). CCR6, thus, may regulate B-1 cell recruitment to PVAT and control local inflammatory responses by secreting IgM and thereby its use in development of cell-based therapeutics could help in modulation of the disease (Figure 6).

## Data Availability Statement

The original contributions presented in the study are included in the article/Supplementary Material, further inquiries can be directed to the corresponding author/s.

## Ethics Statement

The studies involving human participants were reviewed and approved by University of Virginia Human Investigation Committee. The patients/participants provided their written informed consent to participate in this study. The animal study was reviewed and approved by University of Virginia Animal Care and Use Committee.

## Author Contributions

PS designed and performed the experiments, acquired and analyzed the data, prepared figures, and wrote the manuscript. AU, HMP, MAM, and CM performed the experiments. FD analyzed human data. SVB edited scientific content in the manuscript. AMT acquired coronary angiography data. CAM designed the experiments and edited the manuscript. All authors contributed to the article and approved the submitted version.

## Conflict of Interest

The authors declare that the research was conducted in the absence of any commercial or financial relationships that could be construed as a potential conflict of interest.

## Supplementary Material

Supplementary Figure 1 B-1 cells do not survive longer periods in sIgM deficient mice: **(A)** schematic representation of experimental design. FACS sorted B-1 cells *ApoE*^−/−^ mice were adoptively transferred into *sIgM*^−/−^*ApoE*^−/−^ mice. After 1 weeks of the transfer, mice were maintained on WD for 6 weeks. **(B)** After blood was collected at different time points and circulatory IgM levels were measured by ELISA (n = 4/group).

Supplementary Figure 2 FACS confirmation of MACS purified CD43^–^ B cells: CD43^–^ splenocyte isolation was performed by MACS separation. CD43^+^ B cells were shown before MACS and after MACS separation.

Supplementary Figure 3 No difference in total cholesterol levels. Serum cholesterol levels were measured in *sIgM*^−/−^*ApoE*^−/−^ mice that received PBS and CD43^–^ splenocytes from different genotypic donor mice followed by 12 weeks of WD feeding (Figure 5A). There was no difference in serum total cholesterol levels between these groups. Results are represented in mean + SEM, Mann-Whitney unpaired *t*-test was performed. n = 8–9 mice/group and each dot represents individual mouse.

Supplementary Figure 4 No difference in circulatory IgG levels. Serum **(A)** total IgG, **(B)** MDA mimotope specific IgG, and **(C)** total IgE levels were measured in *sIgM*^−/−^*ApoE*^−/−^ mice that received PBS and CD43^–^ splenocytes from different genotypic donor mice followed by 12 weeks of WD feeding (Figure 5A). There was no difference between control (PBS) group and CD43^–^ splenocytes transferred from different genotypic mice groups. Results are represented in mean + SEM, Mann-Whitney unpaired *t*-test was performed. n = 8–9 mice/group and each dot represents individual mouse.

Supplementary Figure 5 Total B cells and few percent of T cells express CCR6 but not monocytes in PBMCs: Flow cytometry analysis to determine CCR6 expression levels on different circulating immune cells in human. CCR6 highly expressed on B cells and moderately expressed on T cell subsets but no expression on monocytes (n = 15).
