# Supplementary figures and images for: Chemokine Receptor-6 Promotes B-1 Cell Trafficking to Perivascular Adipose Tissue, Local IgM Production and Atheroprotection
Source: Front Immunol. 2021 Feb 19;12:636013. doi: 10.3389/fimmu.2021.636013 (PMC7933012; doi:10.3389/fimmu.2021.636013)

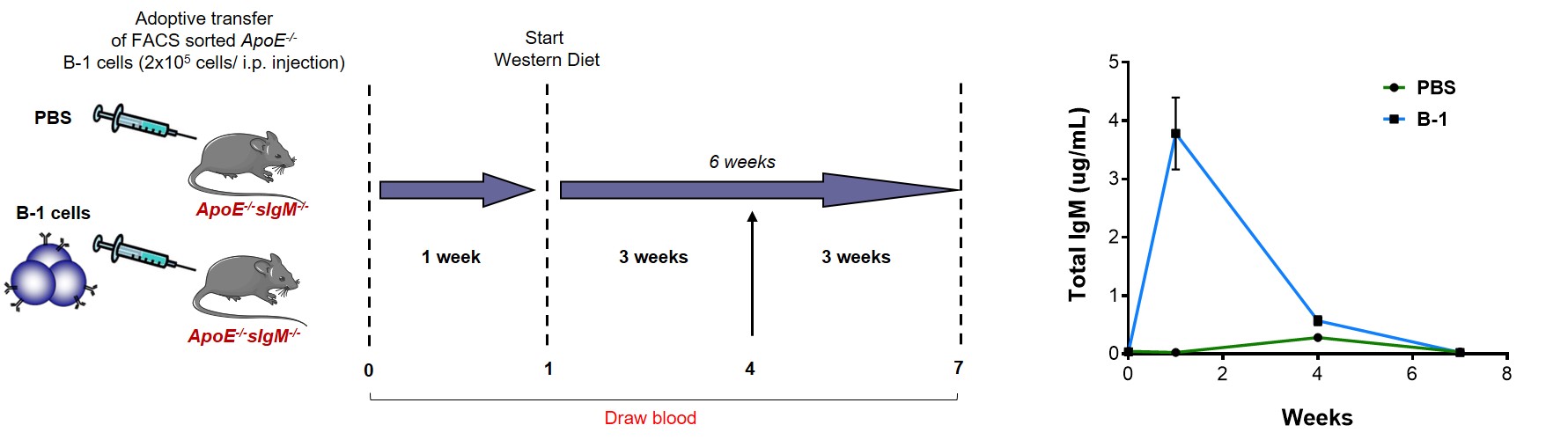

Supplement: Supplementary Figure 1 — B-1 cells do not survive longer periods in sIgM deficient mice: (A) schematic representation of experimental design. FACS sorted B-1 cells ApoE−/− mice were adoptively transferred into sIgM−/−ApoE−/− mice. After 1 weeks of the transfer, mice were maintained on WD for 6 weeks. (B) After blood was collected at different time points and circulatory IgM levels were measured by ELISA (n = 4/group). [file Image_1.JPEG]

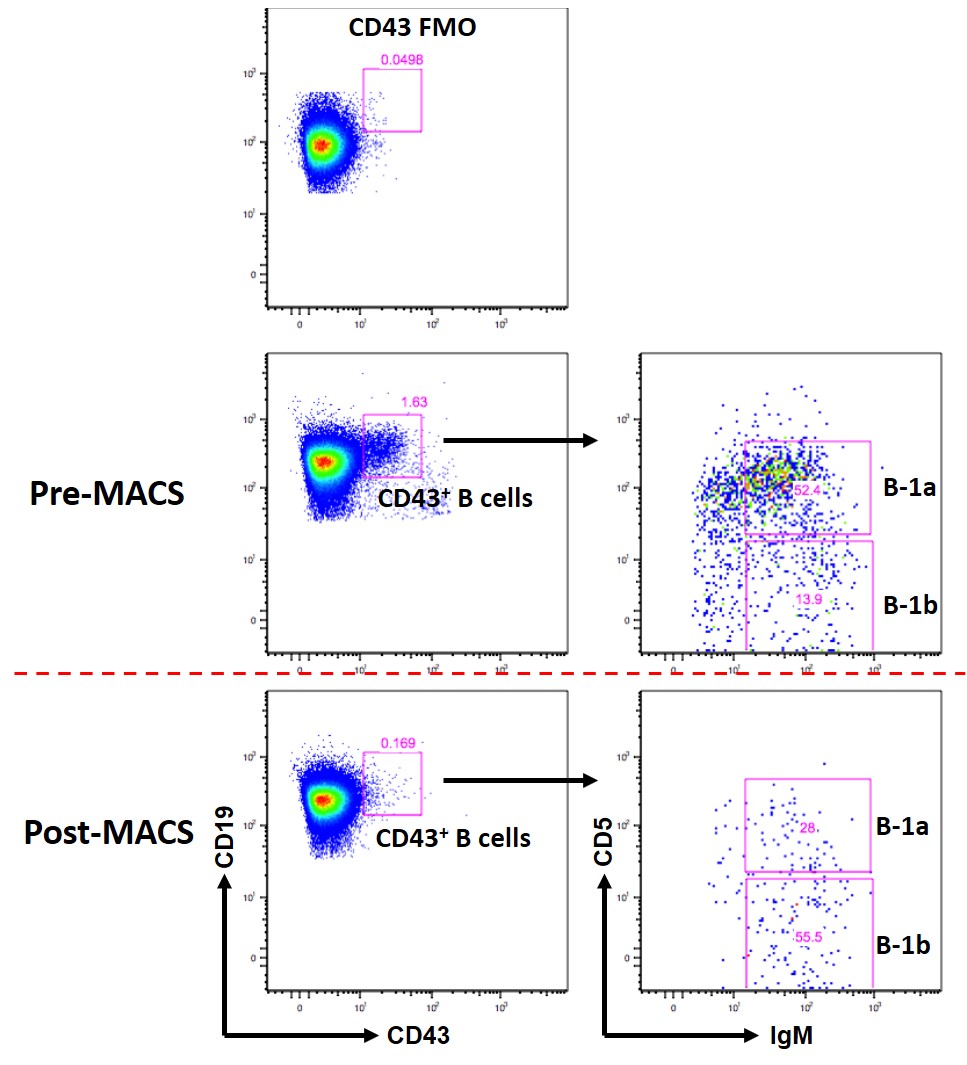

Supplement: Supplementary Figure 2 — FACS confirmation of MACS purified CD43– B cells: CD43– splenocyte isolation was performed by MACS separation. CD43+ B cells were shown before MACS and after MACS separation. [file Image_2.JPEG]

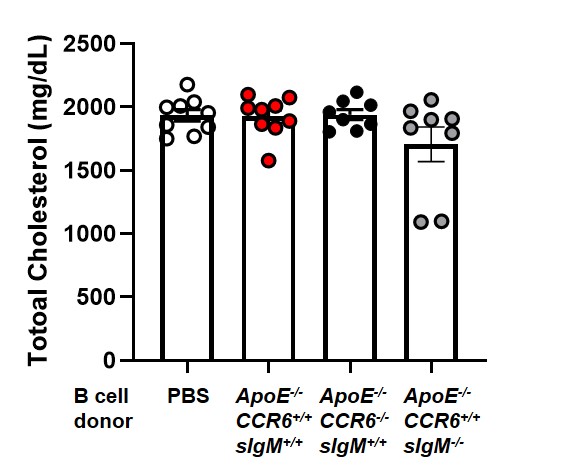

Supplement: Supplementary Figure 3 — No difference in total cholesterol levels. Serum cholesterol levels were measured in sIgM−/−ApoE−/− mice that received PBS and CD43– splenocytes from different genotypic donor mice followed by 12 weeks of WD feeding (Figure 5A). There was no difference in serum total cholesterol levels between these groups. Results are represented in mean + SEM, Mann-Whitney unpaired t-test was performed. n = 8–9 mice/group and each dot represents individual mouse. [file Image_3.JPEG]

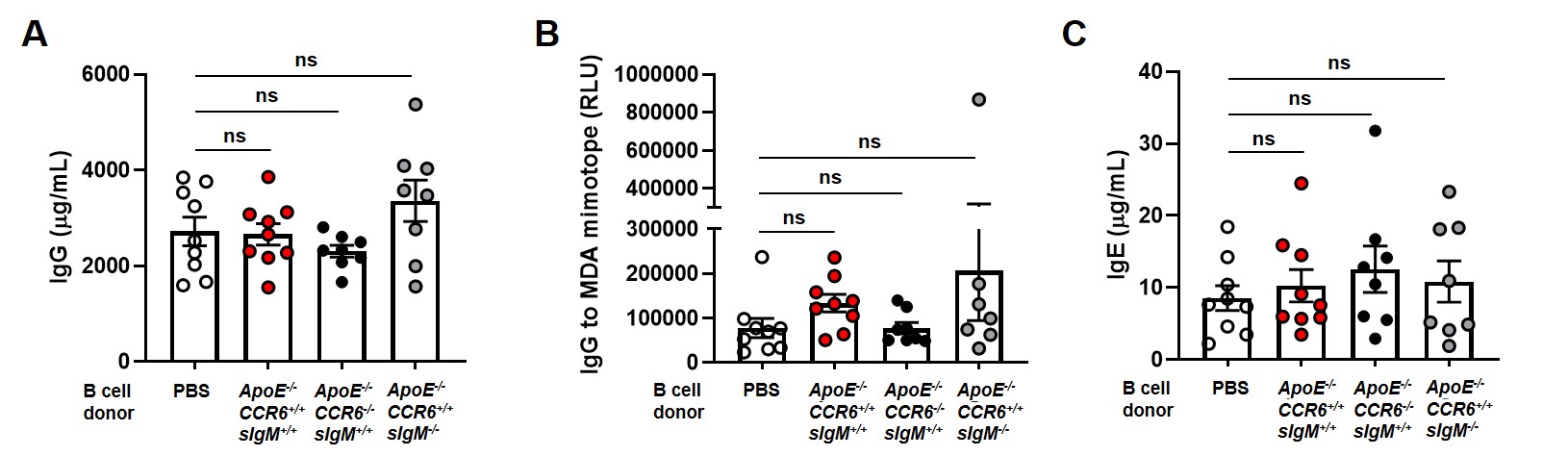

Supplement: Supplementary Figure 4 — No difference in circulatory IgG levels. Serum (A) total IgG, (B) MDA mimotope specific IgG, and (C) total IgE levels were measured in sIgM−/−ApoE−/− mice that received PBS and CD43– splenocytes from different genotypic donor mice followed by 12 weeks of WD feeding (Figure 5A). There was no difference between control (PBS) group and CD43– splenocytes transferred from different genotypic mice groups. Results are represented in mean + SEM, Mann-Whitney unpaired t-test was performed. n = 8–9 mice/group and each dot represents individual mouse. [file Image_4.JPEG]

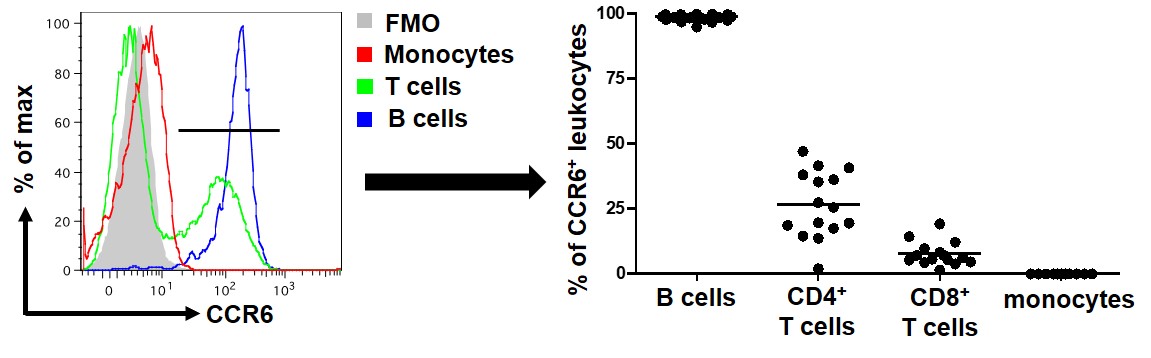

Supplement: Supplementary Figure 5 — Total B cells and few percent of T cells express CCR6 but not monocytes in PBMCs: Flow cytometry analysis to determine CCR6 expression levels on different circulating immune cells in human. CCR6 highly expressed on B cells and moderately expressed on T cell subsets but no expression on monocytes (n = 15). [file Image_5.JPEG]
