# Supplementary material for: Chemokine Receptor-6 Promotes B-1 Cell Trafficking to Perivascular Adipose Tissue, Local IgM Production and Atheroprotection
Source: Front Immunol. 2021 Feb 19;12:636013. doi: 10.3389/fimmu.2021.636013 (PMC7933012; doi:10.3389/fimmu.2021.636013)
Supplement: Supplementary file 6 [file Table_1.DOCX]

**Supplement table 1:** Characteristics of coronary artery disease patients

| **Variable - Mean [IQR], n (%)** | **GS low (n=80)** | **GS high (n=38)** | **p-value** |
| --- | --- | --- | --- |
| **Age** | 62 [13] | 63 [15] | 0.25 |
| **Sex (Female)** | 36 (45%) | 12 (68%) | 0.17 |
| **Ethnicity (Non-Hispanic)** | 79 (99%) | 37 (100%)* | 0.49 |
| **Race (Caucasian)** | 76 (95%) | 36 (95%) | 0.95 |
| **Hypertension** | 65 (81%) | 30 (79%) | 0.77 |
| **Diabetes** | 25 (31%) | 19 (50%) | 0.05 |

*missing = 1

GS: Gensini score
